# Zinc Promotes Mitochondrial Health Through PGC-1alpha Enhancing Bacterial Clearance in Macrophages Infected with *Mycobacterium avium Complex*

**DOI:** 10.3390/ijms26199270

**Published:** 2025-09-23

**Authors:** Ruxana T. Sadikot, Prabagaran Narayanasamy, Zhihong Yuan, Deandra Smith, Daren L. Knoell

**Affiliations:** 1 VA Nebraska Western Iowa Health Care System, Omaha, NE 68105, USA; 2 Division of Pulmonary, Critical Care & Sleep, Department of Internal Medicine, University of Nebraska Medical Center, Omaha, NE 68198, USA; p.narayanasamy@unmc.edu (P.N.); zhyuan@unmc.edu (Z.Y.); deandra.smith@unmc.edu (D.S.); 3 Department of Pharmacy and Science, University of Nebraska Medical Center, Omaha, NE 68198, USA

**Keywords:** mitochondria dysfunction, *Mycobacterium avium*, biogenesis, zinc, macrophages

## Abstract

Mitochondria are increasingly recognized as important contributors to immune function, in addition to energy production. They exert this influence through modulation of various signaling pathways that regulate cellular metabolism and immune function in response to pathogens. Peroxisome proliferator-activated receptor (PPAR) coactivator 1 alpha (PGC-1α) is the primary transcription factor and regulator involved in mitochondrial biogenesis. Long known to be involved in immune function, zinc (Zn) is also required for proper mitochondrial function. It is increasingly recognized that many cellular immunometabolic activities are also Zn-dependent. Taken together, we investigated the role of Zn deficiency, both dietary and genetically induced, and Zn supplementation in PGC-1α-mediated macrophage mitochondrial biogenesis and immune function following infection with *Mycobacterium avium complex* (MAC). Our novel findings show that Zn is an important regulator of PGC-1α, TFAM and mitochondrial biogenesis, leading to enhanced bacterial phagocytosis and bacterial killing in macrophages. Mechanistically, we show that the Zn importer ZIP8 (Zrt/Irt-like protein) orchestrates Zn-mediated effects on PGC-1α and mitochondrial function. Taken together, defective Zn biodistribution may increase susceptibility to infection, whereas Zn supplementation may provide a tractable host-directed therapy to enhance the innate immune response in patients vulnerable to MAC infection.

## 1. Introduction

The human immune system is constantly under siege from microbes and pathogens. The respiratory tract, due to constant interaction with airborne pathogens, is especially vulnerable to bacterial exposure and, as such, has developed multiple lines of defense to prevent infection. Among the different immune cells that respond to pathogens in the lungs, tissue-resident alveolar macrophages are the front-line responders to inhaled pathogens. Alveolar macrophages play a vital role in host defense and homeostasis, as they phagocytose microbes and cellular debris within the fragile alveolar compartment. This task requires a high level of energy demand and requires proper mitochondrial function for a robust yet balanced response that removes bacteria and restores order. The transcription factor Peroxisome proliferator-activated receptor (PPAR) coactivator 1 alpha (PGC-1α) is the primary energetic regulator that drives mitochondrial biogenesis and turnover. PGC-1α function is influenced through the activity of other transcription factors, including mitochondrial transcription factor A (TFAM) and transcription factor EB (TFEB), in addition to critical cofactors, paramount among them elemental zinc (Zn). This study, for the first time, investigates the role of PGC-1α and the contribution of zinc to regulate the factors of mitochondrial function in macrophage immune response.

The atypical transition metal Zn is an indispensable component of many physiological processes, including immunity and metabolism, with over 3000 different proteins and enzymes reliant upon Zn for functional or catalytic activity [1,2,3]. It is well established that Zn is essential for proper immune system function, with dietary-induced deficiency resulting in elevated inflammation and impaired responses to pathogens and excess causing cytotoxicity and cell death [4]. Accordingly, the transport of Zn in and out of cells is tightly regulated to maintain a narrow window of homeostatic concentrations [2,5,6,7,8,9,10]. The primary regulators of intracellular zinc levels are transmembrane transporters belonging to two different families that control intracellular Zn import through Zrt-Irt protein (ZIPs^1–14^) and export through ZnTs^1–10^. ZIPs and ZnTs function in a coordinated manner to shuttle Zn into and out of cells to always maintain essential cellular function. Here, we investigate how the zinc transporter ZIP8 influences macrophage host defense, particularly as it relates to mitochondrial biogenesis.

Due to high energy requirements that drive host defense, immune cells are highly reliant upon efficient mitochondrial function, which is directly coupled to access to available intracellular Zn. Thus, Zn transport into and out of cells is tightly regulated and a highly controlled process [11]. Deficits in cytosolic Zn through ZIPs or excess accumulation due to defective export through ZnTs can also adversely impact mitochondrial function. Among the Zn-importing family members, our group was the first to reveal that ZIP8 is indispensable in myeloid cell function in response to bacterial infection [12]. In particular, ZIP8 expression is rapidly induced upon infection and mobilizes Zn from the vasculature into the cytosol, where it then serves a number of functions, resulting in a robust yet balanced proinflammatory response [1]. Loss of ZIP8 expression in bone marrow-derived macrophages and dendritic cells results in a significant reduction in available Zn, an overly exuberant inflammatory response, and reduced bacterial clearance [13]. However, the mechanisms by which Zn and Zn transporters affect mitochondrial homeostasis in infection have not been determined.

Nontuberculous mycobacteria (NTM) refer to mycobacteria other than *Mycobacterium tuberculosis* (M.tb) and *M. leprae*. NTM are Gram-positive, acid-fast aerobic bacilli that are ubiquitous in the environment and can be normal inhabitants of natural and drinking water systems, pools, hot tubs, bird droppings, dust, milk, soil, and even laboratory equipment. The incidence and prevalence of NTM-related disease are rising at an alarming rate worldwide. This increase has been attributed to environmental and climate changes, genetic mutations, nosocomial infections, and improved molecular testing. Of significant concern is increasing rates of antibiotic resistance and morbidity associated with prolonged infection, especially in the elderly and non-smoking women. Chronic pulmonary bacterial infections place a heavy immunological burden on lung cells, due to the prolonged and often unrecognized course of infection. *Mycobacterium avium complex* (MAC) is a species of nontuberculous mycobacterium (NTM) that is present in the environment that humans frequently encounter. Patients with immunodeficiencies, including those with HIV, COPD, and cystic fibrosis, or those who use immunosuppressant agents, are at an elevated risk for pathogenic infection and pulmonary complications [14,15,16,17].

Current treatment protocols involve the use of multiple antibiotics for extended intervals, which are becoming less effective due to the emergence of drug-resistant strains. Because of this, alternative treatment options are needed. A relatively new paradigm has emerged that aims to enhance the bactericidal activity of immune cells through host-directed therapies (HDTs). Increasing the activity and effectiveness of an individual’s immune cells has the potential to circumvent antibiotic resistance, resulting in improved outcomes. We hypothesize that enhancing mitochondrial function will enhance macrophage immune function to clear NTM, and we will investigate the role of Zn on mitochondrial biogenesis and bacterial clearance [16].

Our previous work was the first to reveal that pharmacological activation of PGC-1α has a positive impact on the ability of macrophages to eradicate MAC and other NTMs [17,18,19]. Herein, we reveal that Zn has a positive impact on the expression and activity of PGC-1α, and that the Zn transporter ZIP8 serves as a vital conduit for the mobilization of Zn into macrophages in response to NTM infection. First, we show the effects of Zn deficiency and Zn supplementation on antibacterial activity in macrophages. We then reveal that Zn serves as an immunomodulator by regulating mitochondrial function through PGC-1α and related cofactors. Expanding from this, we investigate the effects of Zn supplementation on macrophages to attenuate MAC-mediated deficits in mitochondrial and immune activity. To investigate the mechanism, we utilize a novel ZIP8-KO mouse model to determine the influence of this specific Zn importer on PGC-1α-mediated immune cell function during MAC infection. Our results show the interplay between Zn concentrations and levels of PGC-1α within macrophages, providing a hitherto uninvestigated link between PGC-1α-mediated mitochondria function in immune cells and cellular zinc concentrations.

## 2. Results

### 2.1. Zn Deficiency Induces Mitochondrial Damage, Increases Mitochondrial ROS Production, and Attenuates Bacterial Clearance

**Figure 1:**
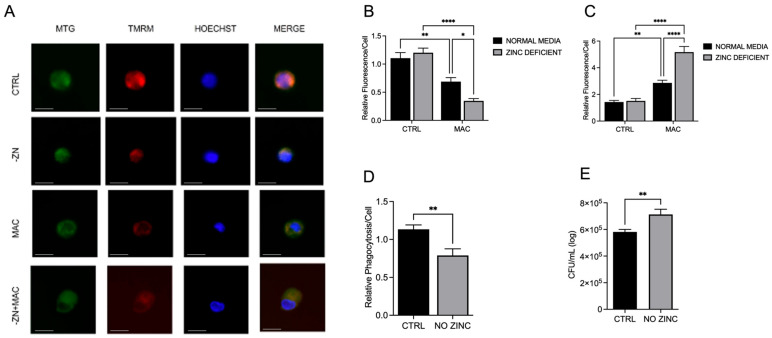
Zinc deficiency during MAC infection damages mitochondria through reduction of ΔΨm and increased mitochondrial ROS production and reduces macrophage phagocytosis and bacterial killing. U937 macrophages were cultured and introduced to zinc-deficient conditions for 48 h, followed by MAC infection (MOI 1) for 24 h prior to all analyses. Cells were stained with MitoTracker Green, tetramethylrhodamine methyl ester (TMRM), and Hoechst to evaluate mitochondrial membrane integrity and presented as relative fluorescence of TMRM/MTG per Hoechst-positive cell (**A**,**B**); scale bar 20 µm. Mitochondria superoxide production was evaluated through staining with MitoSOX and measuring fluorescence expression within cells (**C**). Phagocytic uptake was determined through incubating cells with fluorescent BioParticles for 2 h and evaluating fluorescence expression to determine cellular uptake (**D**). Bacterial killing was evaluated through CFU counting on 7H9 agar plates (**E**). Results are averages of experiments performed in triplicate with SEM. * *p* < 0.05, ** *p* < 0.01, **** *p* < 0.001.

To determine the effect of Zn deficiency on mitochondrial health in MAC-infected macrophages, we evaluated ΔΨm and mitochondrial ROS production. MAC infection significantly reduced the integrity of the mitochondrial membrane potential, which was exacerbated by Zn deficiency (Figure 1A,B). Damage to the mitochondria can result in excess production of reactive oxygen species [20], which can leak from the electron transport chain (ETC) into the surrounding intracellular environment. ROS superoxide production was evaluated through fluorescent expression of MitoSOX. Macrophages infected with MAC exhibited a significant elevation in MitoSOX fluorescence, indicative of excessive production of superoxide, which was significantly increased under Zn-deficient conditions (Figure 1C). Next, we investigated the influence of Zn deficiency on the antibacterial activity of macrophages through evaluating phagocytic uptake and killing of ingested microbes. U937 macrophages were cultured under Zn-deficient conditions for 48 h and incubated with opsonized fluorescent E. coli BioParticles (MOI 2) for 2 h. After washing to remove extracellular bacteria, fluorescence was measured on a microplate reader (480 nm ex/520 nm em) to compare the relative amounts of phagocytic uptake. Zn deficiency resulted in significantly lower phagocytosis compared to Zn-sufficient controls (Figure 1D). The ability of macrophages to kill internalized MAC was evaluated by quantification of colony-forming units (CFUs). U937 macrophages were cultured under normal or Zn-deficient conditions for 48 h, infected with MAC (MOI 1) for 4 h, then incubated with gentamycin-containing medium to kill extracellular bacteria. Cells were lysed 24 h post-infection, and the lysate was added to 7H9 agar plates for the quantification of CFUs. Zn deficiency resulted in significantly higher CFUs compared to control cells, indicative of an elevated bacterial burden and reduced ability to kill MAC (Figure 1E). These data indicate that Zn deficiency causes reduced phagocytosis; however, bacteria that are ingested accumulate over time due to compromised intracellular MAC clearance.

### 2.2. Zinc Deficiency During MAC Infection Reduces Expression of Key Transcriptional Regulators of Mitochondrial Biogenesis

**Figure 2:**
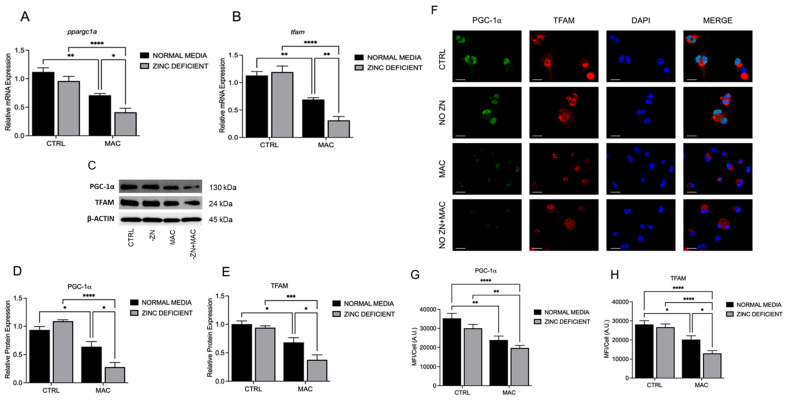
Zinc deficiency during MAC infection reduces expression of key transcriptional regulators of mitochondrial biogenesis: U937 macrophages were cultured and introduced to zinc-deficient conditions for 48 h prior to MAC infection (MOI 1) for 24 h. PCR results for mRNA expression of *ppargc1a* (**A**) and *tfam* (**B**). Western blot results for PGC-1α (**D**) and TFAM (**E**), along with representative blot images (**C**). ICC results for protein expression of PGC-1α (**G**), TFAM (**H**), and representative confocal images (**F**); scale bar 20 µm. Results are averages of experiments performed in triplicate with SEM. * *p* < 0.05, ** *p* < 0.01, *** *p* < 0.005, **** *p* < 0.001.

Zn is known to modulate the activity of multiple transcription factors and coactivators; thus, we first sought to determine whether Zn deficiency adversely affects mitochondrial biogenesis. PGC-1α and TFAM are key transcriptional regulators of mitochondrial biogenesis and determine mitochondrial health and function, which in turn affect macrophage antibacterial function. U937 macrophages were cultured in Zn-deficient conditions for 48 h prior to and then 24 h after MAC infection (MOI 1). Cells cultured in Zn-sufficient medium had reduced *ppargc1a* and *tfam* mRNA expression at 24 h post-infection (HPI). However, Zn deficiency resulted in similar but even lower basal and post-infection mRNA expression levels of *ppargc1a* (Figure 2A) and *tfam* (Figure 2B). Similarly, protein expression exhibited the same trend, with Zn deficiency further reducing PGC-1α (Figure 2C,D) and TFAM (Figure 2C,E) following MAC infection. Protein expression was also evaluated immunohistochemically (ICC) and also showed that Zn deficiency reduced PGC-1α (Figure 2F,G) and TFAM (Figure 2F,H), which was further significantly suppressed following MAC infection. Together, these data show that Zn deficiency significantly attenuates the expression of key regulators of mitochondrial biogenesis that likely impact mitochondrial function.

### 2.3. ZP Treatment Restores PGC-1α and TFAM Expression Following MAC Infection

**Figure 3:**
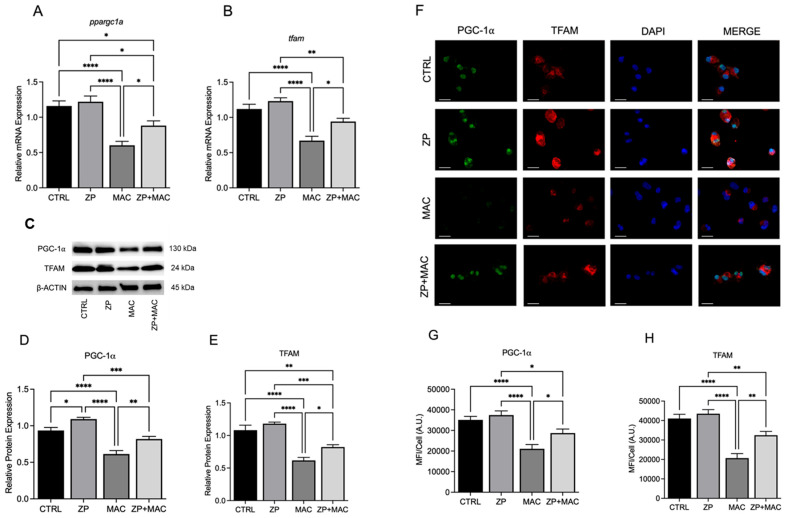
ZP treatment rescues expression of mitochondrial PGC-1α and related cofactors during MAC infection: U937 macrophages were cultured and treated with ZP [50 µM] for 48 h prior to infection with MAC (MOI 1) for 4 h prior to all analyses. PCR results for mRNA expression of *ppargc1a* (**A**) and *tfam* (**B**). Western blot results for PGC-1α (**D**) and TFAM (**E**), along with representative blot images (**C**). ICC results for protein expression of PGC-1α (**G**), TFAM (**H**), and representative confocal images (**F**); scale bar 20 µm. Results are averages of experiments performed in triplicate with SEM. * *p* < 0.05, ** *p* < 0.01, *** *p* < 0.005, **** *p* < 0.001.

While our previous results demonstrate the negative impact of Zn deficiency during MAC infection on PGC-1α and TFAM, we sought to determine whether Zn supplementation can restore their expression. ZinPRO^®^ (ZP), a 1:1 Zn-lysine, Zn-glutamine complex, is an organic, commercially available Zn supplement. U937 macrophages were cultured with ZP [50 μM] for 48 h and infected with MAC (MOI 1) for 24 h, prior to collection for mRNA and protein analysis. Treatment with ZP increased *ppargc1a* (Figure 3A) and *tfam* mRNA expression (Figure 3B), resulting in expression levels significantly higher compared to MAC infection alone. Protein expression exhibited a similar trend, with MAC infection significantly decreasing expression of PGC-1α (Figure 3C,D) and TFAM (Figure 3C,E). ZP treatment significantly increased PGC-1α (Figure 3C,D) and TFAM (Figure 3C,E). Protein expression was also evaluated and visualized through immunohistochemistry (ICC) and confocal microscopy, with results in agreement with the Western blots. MAC infection significantly reduced fluorescence expression of both PGC-1α (Figure 3F,G) and TFAM (Figure 3F,H). Again, pretreatment with ZP abrogated the MAC-mediated decreases in PGC-1α (Figure 3F,G) and TFAM (Figure 3F,H). Treatment with ZP also resulted in significantly elevated PGC-1α expression compared to untreated controls (Figure 3F,G). Together, these results indicate that zinc supplementation restores the expression of transcriptional regulators of mitochondrial biogenesis, thus enhancing mitochondrial function in response to MAC infection.

### 2.4. ZP Treatment Reduces Mitochondrial Damage, Decreases Mitochondrial ROS Production, and Improves Bacterial Clearance

**Figure 4:**
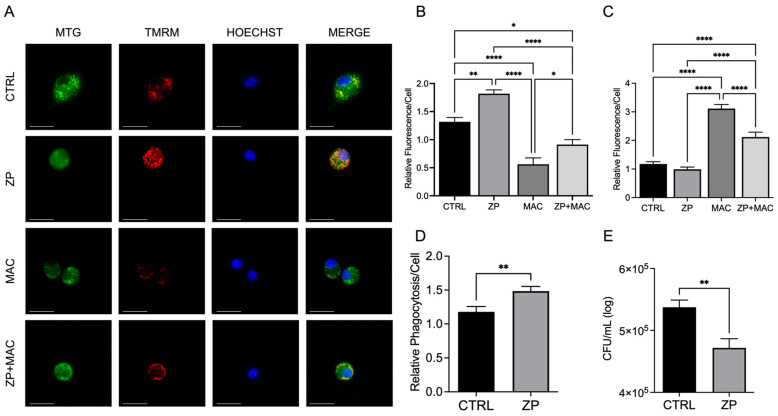
Treatment with ZP aids in maintaining ΔΨm during MAC infection, reduces mitochondrial ROS production, and elevates macrophage antibacterial activity. U937 macrophages were cultured and treated with ZP [50 µM] for 48 h prior to infection with MAC (MOI 1) for 24 h prior to all analyses. Cells were stained with MitoTracker Green, TMRM, and Hoechst to evaluate mitochondrial membrane integrity and presented as relative fluorescence of TMRM/MTG per Hoechst-positive cell (**A**,**B**); scale bar 20 µm. Mitochondria superoxide production was evaluated through staining with MitoSOX and measuring fluorescence expression within cells (**C**). Phagocytic uptake was determined through incubating cells with fluorescent BioParticles for 2 h and evaluating fluorescence expression to determine cellular uptake (**D**). Bacterial killing was evaluated through CFU counting on 7H9 agar plates (**E**). Results are averages of experiments performed in triplicate with SEM. * *p* < 0.05, ** *p* < 0.01, **** *p* < 0.001.

Since ZP treatment improved PGC-1α, TFAM mRNA, and protein expression in response to MAC infection, we next determined the impact of ZP on mitochondrial health and macrophage immune function (Figure 4A,B). We utilized MitoSOX to evaluate superoxide production during MAC infection, as described above. U937 macrophages were pretreated with ZP [50 μM] for 48 h, followed by MAC (MOI 1) infection for 4 h, and MitoSOX was measured by fluorescence. In agreement with our previous results, MAC infection significantly increased superoxide production compared to controls (Figure 4C). ZP treatment did not exhibit a significant difference in MitoSOX fluorescence compared to control but significantly attenuated superoxide production in response to MAC infection (Figure 4C). We next evaluated the influence of ZP on macrophage phagocytic uptake. Compared to control, ZP-treated macrophages exhibited a significant increase in bacterial uptake compared to untreated controls (Figure 4D). To determine the effect of ZP on bactericidal activity of macrophages, cells were pretreated with ZP [50 μM] prior to infection (MOI 1) for 4 h, then lysed and plated on 7H9 agar to determine CFU counts. The ZP-treated macrophages exhibited elevated intracellular killing of MAC, resulting in a significantly lower number of colonies compared to controls (Figure 4E). These results demonstrate that ZP treatment helps to maintain mitochondrial membrane potential and reduce mitochondrial damage, resulting in enhanced macrophage bacterial clearance.

### 2.5. ZIP8 Loss Reduces Expression of Key Transcriptional Regulators of Mitochondrial Biogenesis in Response to MAC Infection

**Figure 5:**
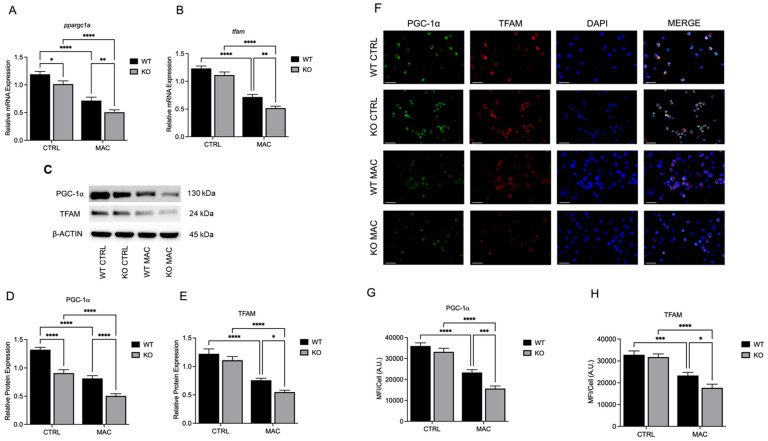
ZIP8-KO reduces expression of key transcriptional regulators of mitochondrial biogenesis in BMDMs. BMDMs from both WT and *Zip8*-KO mice were cultured in normal medium and infected with MAC (MOI 1) for 24 h prior to all analyses. PCR results for mRNA expression of *ppargc1a* (**A**) and *tfam* (**B**). Western blot results for PGC-1α (**D**) and TFAM (**E**), along with representative blot images (**C**). ICC results for protein expression of PGC-1α (**G**), TFAM (**H**), and representative confocal images (**F**); scale bar 50 µm. Results are averages of experiments performed in triplicate with SEM. * *p* < 0.05, ** *p* < 0.01, *** *p* < 0.005, **** *p* < 0.001.

We have previously reported that ZIP8 is the only Zn transporter that is highly induced following bacterial infection and regulates myeloid cell function in a Zn-dependent manner [13]. Therefore, we sought to investigate the role of ZIP8 following MAC infection, as it relates to PGC-1α, TFAM, and mitochondrial biogenesis. Cells were harvested from the bone marrow of WT (control) and ZIP8KO mice and differentiated as previously described [13]. BMDMs were then cultured under Zn-deficient conditions for 48 h, followed by MAC infection (MOI 1) for 24 h. WT and KO BMDMs had similar mRNA expression of *ppargc1a* (Figure 5A) and *tfam* (Figure 5B) under control conditions; however, MAC infection significantly decreased mRNA and protein expression of both factors in the ZIP8KO cultures compared to the WT counterparts (Figure 5A,B and 5C,D respectively). The differences in TFAM expression between the WT and KO cells were less pronounced, with the control cells not exhibiting a significant difference (Figure 5C,E). This trend was also observed using ICC, with significantly reduced fluorescence expression of PGC-1α and TFAM in the KO cells compared to the WT counterparts (Figure 5F–H). These results provide evidence that intracellular Zn deficiency as a consequence of ZIP8 loss also reduces the expression of key mitochondrial transcription regulators, thus exacerbating mitochondrial dysfunction.

### 2.6. ZIP8 Loss Enhances MAC-Induced Mitochondrial Damage, Increased Mitochondrial ROS Production, and Decreased Bacterial Clearance

**Figure 6:**
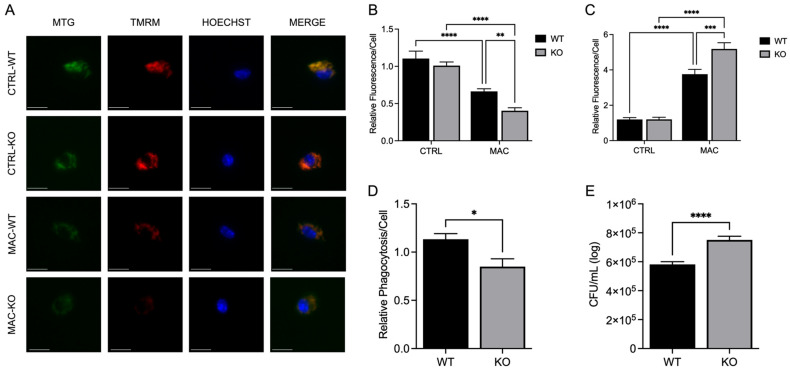
ZIP8 KO BMDMs exhibit reduced ΔΨm, increased mitochondrial ROS production, and reduced macrophage antibacterial activity under zinc deficiency during MAC infection. BMDMs from both WT and *Zip8*-KO mice were cultured in normal medium and infected with MAC (MOI 1) for 4 h prior to all analyses. Cells were stained with MitoTracker Green, TMRM, and Hoechst to evaluate mitochondrial membrane integrity and presented as relative fluorescence of TMRM/MTG per Hoechst-positive cell (**A**,**B**); scale bar 20 µm. Mitochondria superoxide production was evaluated through staining with MitoSOX and measuring fluorescence expression within cells (**C**). Phagocytic uptake was determined through incubating cells with fluorescent BioParticles for 2 h and evaluating fluorescence expression to determine cellular uptake (**D**). Bacterial killing was evaluated through CFU counting on 7H9 agar plates (**E**). Results are averages of experiments performed in triplicate with SEM. * *p* < 0.05, ** *p* < 0.01, *** *p* < 0.005, **** *p* < 0.001.

Knowing that ZIP8KO loss resulted in significantly reduced PGC-1α expression, we determined whether this corresponded with impairment of mitochondrial health and antibacterial activity in BMDMs. We first determined mitochondrial health via the impact on ΔΨm and energy production. WT and KO BMDMs were infected with MAC (MOI 1) for 4 h. Cells were then incubated with MTG and TMRM and subjected to confocal microscopy. No significant differences were observed between the WT and KO cells under control conditions (Figure 6A). However, MAC infection induced a significant decrease in TMRM fluorescence in both WT and KO BMDMs, with the KO cells exhibiting larger decrease compared to the WT. This also corresponded with a substantial decrease in fluorescence expression in the KO cells compared to their WT counterparts (Figure 6A). We did not observe differences in mitochondrial ROS formation under control conditions (Figure 6B). As anticipated, MAC infection increased ROS production in both WT and KO BMDMs, with the KO cells exhibiting significantly higher ROS production compared to the WT cultures, with KO cells exhibiting a nearly 7-fold increase in superoxide production compared to the control WT cells (Figure 6C). These data provide evidence that ZIP8 loss substantially increases mitochondrial ROS production and mitochondrial damage in response to MAC infection. To determine whether it may influence the antibacterial activity of macrophages, we compared phagocytic uptake and intracellular killing of MAC in WT and KO BMDMs. Similar to BMDMs under Zn-deficient conditions, ZIP8KO cells exhibited a significant decrease in phagocytic uptake compared to the WT (Figure 6D). In addition, similar to Zn-deficient conditions, the KO cells exhibited a significant increase in CFUs compared to the WT cultures (Figure 6E), indicative of a higher bacterial burden despite reduced phagocytic uptake.

### 2.7. ZP Treatment Restores PGC-1α and TFAM Expression Following MAC Infection of ZIP8KO BMDMs

**Figure 7:**
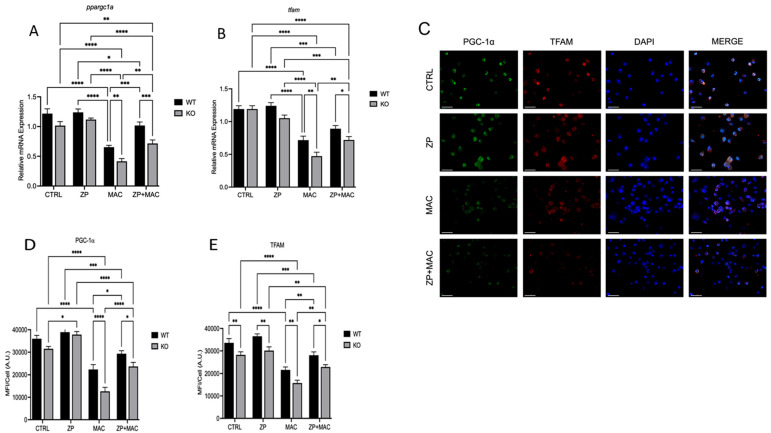
ZP treatment rescues ZIP8 KO BMDM expression of mitochondrial PGC-1α and related cofactors during MAC infection. BMDMs from both WT and *Zip8*-KO mice were cultured in normal medium and treated with ZP [50 µM] for 48 h, followed by infection with MAC (MOI 1) for 4 h prior to all analyses. PCR results for mRNA expression of *ppargc1a* (**A**) and *tfam* (**B**). ICC results for protein expression of PGC-1α (**D**), TFAM (**E**), and representative confocal images (**C**); scale bar 50 µm. Results are averages of experiments performed in triplicate with SEM. * *p* < 0.05, ** *p* < 0.01, *** *p* < 0.005, **** *p* < 0.001.

Human studies have revealed that a frequently occurring ZIP8 variant allele can lead to defective intracellular transport of Zn and is strongly associated with bacterial infections (Ye 2014 [21]). In our previous work, we revealed that Zn pyrithione, which obviates the need for ZIP-mediated transport, can restore host defense in ZIP8KO macrophages against *S. pneumoniae* infection (Hall et al., 2021 [13]). Thus, we determined whether ZP supplementation, which also does not require ZIP-mediated transport, can restore antibacterial function in ZIP8 KO BMDMs. WT and KO BMDM cultures were treated with ZP [50 μM] for 48 h and infected with MAC (MOI 1) for 4 h prior to analysis for mRNA and protein expression. mRNA expression of *ppargc1a* was reduced in the KO cells compared to the WT controls, and ZP treatment resulted in a modest increase in both WT and KO BMDMs (Figure 7A). As previously observed, MAC infection significantly reduced *ppargc1a* expression in both WT and KO cells, with the latter exhibiting a more drastic decrease. However, ZP treatment was able to attenuate this decrease. KO BMDMs treated with ZP and infected with MAC exhibited a significant increase in *ppargc1a* (Figure 7A) and *tfam* (Figure 7B) mRNA expression compared to infection alone. Protein expression was evaluated by immunohistochemistry (Figure 7C–E). This corresponded with a significant increase in PGC-1α and TFAM, which was more significantly elevated in KO cells with ZP treatment compared to WT control cultures (Figure 7D,E). These results reveal that ZP supplementation prior to and during MAC infection restores mitochondrial biogenesis despite ZIP8 loss, which may have tractable clinical implications in the treatment of NTM disease.

### 2.8. ZP Attenuates Mitochondrial Damage and Deficits in Macrophage Antibacterial Activity During MAC Infection in ZIP8 Knockout Macrophages

**Figure 8:**
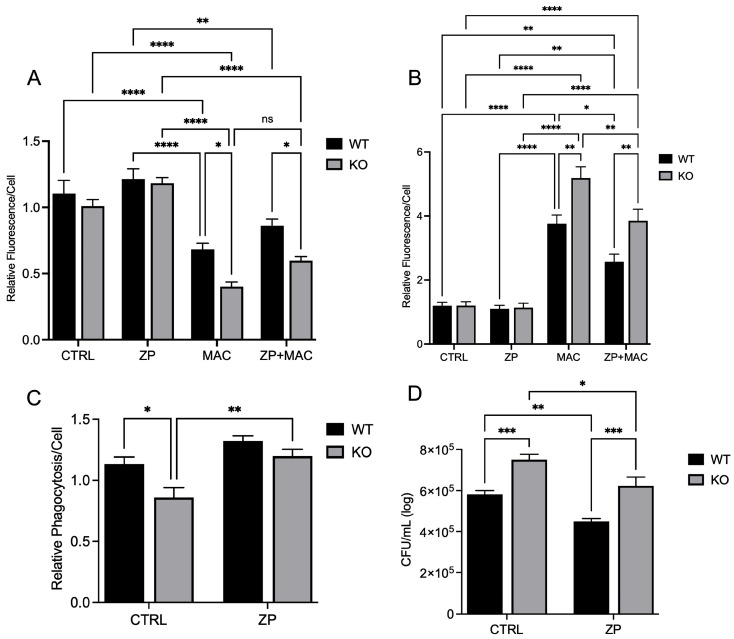
ZP attenuates ZIP8 KO BMDM mitochondrial damage and deficits in macrophage activity during MAC infection. BMDMs from both WT and *Zip8*-KO mice were cultured in normal medium and treated with ZP [50 µM] for 48 h, followed by infection with MAC (MOI 1) for 4 h prior to all analyses. Cells were stained with MitoTracker Green, TMRM, and Hoechst to evaluate mitochondrial membrane integrity and presented as relative fluorescence of TMRM/MTG per Hoechst-positive cell (**A**). Mitochondria superoxide production was evaluated through staining with MitoSOX and measuring fluorescence expression within cells (**B**). Phagocytic uptake was determined through incubating cells with fluorescent BioParticles for 2 h and evaluating fluorescence expression to determine cellular uptake (**C**). Bacterial killing was evaluated through CFU counting on 7H9 agar plates (**D**). Results are averages of experiments performed in triplicate, with SEM. * *p* < 0.05, ** *p* < 0.01, *** *p* < 0.005, **** *p* < 0.001.

Next, we determined whether ZP treatment could reduce mitochondrial damage and improve antibacterial activity despite ZIP8 loss. As before, we evaluated mitochondrial membrane integrity and ROS production under identical conditions. We observed no significant difference between controls and ZP-treated cultures at baseline. MAC infection resulted in a significant increase in TMRM fluorescence in both WT and KO cultures (Figure 8A). ZP treatment improved mitochondrial membrane potential following MAC infection in both WT and KO cells, as seen by significant increases in fluorescence compared to infection alone. KO BMDMs appeared to be more sensitive to mitochondrial damage during MAC infection, as they exhibited significantly less fluorescence expression compared to their WT counterparts (Figure 8A). Differences in ROS production were observed in WT and KO BMDMs following MAC infection, with KO cells exhibiting a more significant increase in superoxide production compared to WT cultures (Figure 8B). ZP treatment resulted in decreased ROS detection in WT and KO cultures in response to MAC infection. Phagocytic uptake was measured as before, with WT and KO BMDMs treated with ZP [50 μM] for 48 h and incubated with fluorescent bacteria for 2 h prior to analysis. As previously observed, the WT cells exhibited an increase in particle uptake compared to the KO cells (Figure 8C). ZP treatment had a positive effect on phagocytic uptake in both WT and KO cells, with both exhibiting significant increases compared to control. As expected, KO BMDMs exhibited a reduced capacity for killing bacteria, resulting in a significantly higher bacterial burden compared to the WT cells (Figure 8D), but this was reversed following ZP treatment, resulting in a significant increase in intracellular killing of MAC in both WT and KO cells, with each cell type exhibiting significantly less bacteria growth than the untreated cells. These results indicate that ZP has mitochondrial protective properties that can overcome loss of ZIP8 and thereby enhance the antibacterial activity of macrophages against MAC infection.

## 3. Discussion

Alveolar macrophages are the primary cells involved in the initial innate immune response against intracellular pathogens [22,23,24,25]. Due to their “first responder” status, alveolar macrophages require a high metabolic rate to eliminate encountered bacteria. High energy demand is predicated upon highly efficient mitochondrial function to power the cellular machinery involved in bactericidal activities [23,24,25,26,27]. Here, we investigated the role of Zn in regulating mitochondrial biogenesis, which is critical for rejuvenating mitochondria for effective ATP production and a robust host immune response. Our data show that Zn enhances the expression of PGC-1α and TFAM, critical transcriptional regulators of mitochondrial biogenesis. Mechanistically, we show that the Zn importer ZIP8 (Zrt/Irt-like protein) regulates Zn-mediated effects on PGC-1a and mitochondrial function.

PGC-1α is a key regulator of mitochondria biogenesis, an important quality control mechanism to maintain the metabolic function of macrophages. Increased expression provides cellular signals required for the maintenance and generation of new mitochondria [28,29,30,31,32]. It is also well established that Zn is required for proper immune function. For the first time, we provide evidence demonstrating a novel Zn-dependent requirement for PGC-1α expression in macrophages in response to MAC infection [2,3,33]. This observation is consistent with other studies demonstrating the essential role of Zn in maintaining lung immune function. Insufficient Zn levels due to restricted dietary intake before respiratory tract infection result in bacterial evasion of the immune system and worse outcomes [2,6,13,34,35,36,37,38].

The acquisition and progression of respiratory tract infections are known to be influenced by chronic Zn deficiency, due to the importance of Zn in a myriad of immune cell functions [2,3]. Populations that are most vulnerable to community-acquired pneumonia also exhibit a high incidence of Zn deficiency [39,40,41], which is estimated to impact approximately 2 billion people worldwide, including approximately 20–30% of the U.S. population [42]. Consistent with this, others have reported that dietary Zn deficiency increases susceptibility to gastrointestinal tract infections [43] and pneumonia [44], whereas the incidence of pneumonia and other infections is decreased with Zn supplementation [45,46,47].

Mice fed Zn-deficient diets were significantly more susceptible to *Streptococcus pneumoniae* infection than their Zn-sufficient counterparts, exhibiting a higher bacterial burden, elevated inflammatory cytokine production, and a drastic decrease in survival [38]. *Acinetobacter baumannii* is a Gram-negative bacterium that is frequently linked to respiratory infections during intubation, resulting in ventilator-associated pneumonia [48]. Zinc deficiency also influenced the progression of *A. baumannii* in vivo in a mouse model, with mice fed a Zn-deficient diet exhibiting a significant bacterial burden in lung tissue and BAL fluid compared to Zn-replete mice [48]. In our previous study investigating infection with *S. pneumoniae*, we utilized a novel *Zip8* KO mouse to determine its effect on immune cell function against infection. Despite being maintained on a sufficient Zn diet, ZIP8 loss in myeloid cells resulted in decreased acquisition of intracellular Zn, as well as increased bacterial burden in the lung, bacterial dissemination into other tissues, increased inflammation and collateral tissue damage, and increased mortality, similar to findings associated with inadequate dietary intake [13]. This has clinical relevance, as human genome-wide association (GWAS) studies revealed that a frequently occurring defective ZIP8 variant allele (rs13107325; Ala391Thr risk allele) is highly associated with inflammation-based diseases [49,50] and bacterial infections [21]. In fact, a comprehensive review of human GWAS studies revealed that the SLC39A8 variant allele is in the top ten of all variant alleles associated with human disease [50]. Collectively, these studies highlight the essential requirement for Zn and its bio redistribution in response to bacterial invasion in order to mount an effective, yet balanced, host innate immune response [51]. Our studies are the first to show the importance of zinc deficiency and supplementation on macrophage function in MAC infection. It is important to recognize that both Zn deficiency and ZIP8 loss had a similar impact on bacterial clearance. ZIP8 loss would decrease importation of Zn from outside the cell into the cytosol. Similarly, ZIP8, which is present on the phagolysosomal membrane would transport Zn out of this compartment and into the cytosol. In other work (under revision), we reveal that ZIP8 loss decreases intracellular Zn content in BMDMs before and after bacterial infection, as measured by inductively coupled plasma mass spectroscopy (ICP-MS). Taken together, it is most likely that decreased intracellular Zn content adversely impacts mitochondrial function, which is vital for proper phagolysosome-mediated removal of intracellular bacteria. However, we cannot completely rule out the possibility that access to Zn by MAC may also play a role.

MAC is ubiquitously present in water sources and soil and can be pathogenic in patients with compromised immune systems and chronic lung diseases, including cystic fibrosis, chronic obstructive pulmonary disease, and bronchiectasis [20,52,53,54,55,56]. MAC infections typically arise from exposure to environmental niches in the water and soil, whereas person-to-person transmission is uncommon [54]. The increasing number of cases is due in part to the emergence of drug-resistant strains, complicating treatment of an already evasive and difficult-to-treat pathogen. Currently, the standard of care is long-term treatment (12–18 months) with multiple antibiotics, which are often difficult to tolerate. Furthermore, a significant number of patients experience recurrence of infection despite prolonged treatment [56]. Thus, there is a high demand for alternative treatment modalities, particularly host-directed therapies that can enhance MAC clearance.

Zn, in addition to other transition metal ions, is also utilized by bacteria for growth and metabolic processes, and immune cells have adapted to this shared need for cations between pathogens through a process known as nutritional immunity [33,57,58]. During infection, metal ions can either be sequestered to prevent their use and uptake by pathogens or concentrated in cellular compartments in excess to induce toxicity in the invading microbes [7,58]. Within the body, Zn possesses secondary messenger functions involved in immune cell recruitment and differentiation. Phagocytic uptake of microbes by macrophages is influenced by the presence of Zn, with ZIP8 expression being rapidly upregulated in response to pathogens to increase intracellular Zn concentrations [1,51]. Zn also influences the activity of other immune cells, with neutrophils exhibiting reduced recruitment, migration, and phagocytosis under conditions of Zn deficiency. Similarly, both mast cells and NK cells utilize Zn to regulate their immune functions, with Zn starvation resulting in deficient immune activity [11]. Importantly, ZIP8 is the only ZIP out of 14 whose expression is significantly increased in myeloid-lineage cells following bacterial recognition, and its import is important in balancing NFκB-mediated signaling. Accordingly, it is plausible that multiple signaling pathways could be involved in mitochondrial-mediated macrophage response to MAC.

Our data show that insufficient intracellular Zn adversely impacts mitochondrial function via reduction in PGC-1α expression in response to MAC. We also reveal that this deficit can be attenuated through Zn supplementation. Instead of supplementing conventional inorganic Zn salts such as ZnCl_2_ or ZnSO_4_, we used a ZinPro^®^ (ZP) formulation in which Zn is conjugated to lysine and glutamine, provided in equal amounts. Previously, it has been shown to have superior bioavailability to inorganic Zn via amino acid transporter-coupled uptake. In a previous study, Lu et al. showed that Zn is essential for transcriptional expression of PGC1 alpha in endothelial cells [59]. We have performed experiments using intracellular zinc depletion using tris(2-pyridylmethyl)amine (TPA), a membrane-permeable zinc chelator which showed similar results on bacterial killing. However, this is the first study to show the expression of PGC-1α in relation to zinc in macrophages infected with MAC. Future studies will investigate the molecular mechanisms of regulation of PGC-1α by zinc.

We provided evidence that the availability of zinc influences PGC-1α expression and sought to determine the mechanism of cellular zinc import. The ZIP family contains 14 members, with variable presence and expression dependent upon tissue location and type. The predominant ZIP in lung tissues is ZIP8, leading us to select it as a potential target influencing PGC-1α expression and related mitochondrial function.

Our previous research investigated the influence of PGC-1α in NTM infections in macrophages, where expression was modulated through treatment with specific activators and inhibitors to determine the effect on macrophage antibacterial activity in nontuberculous mycobacterium (NTM) infections [17]. PGC-1α is a transcription factor and master regulator of mitochondrial biogenesis, influencing the creation and turnover of mitochondria. Functioning in concert with PGC-1α, mitochondrial transcription factor A (TFAM) is responsible for the maintenance and synthesis of mitochondrial DNA (mtDNA), influencing the activity and health of mitochondria [60]. Within the scope of immunity, mitochondrial function drives immune cell activity and function through energy production and metabolism, in addition to important cellular signaling activities. The process of pathogen clearance that includes initial recognition, phagocytic uptake, and eventual lysosomal degradation requires a high energetic demand and metabolic input. Thus, mitochondria are indispensable for protection against bacteria and other pathogens. Revealing that PGC-1α is reliant upon Zn for proper expression and activity provides a new paradigm that involves dietary intake, or lack thereof, and proper biodistribution, via improved formulation and/or ZIP-mediated transport and immunometabolism, resulting in effective clearance of MAC by macrophages. One of the major limitations of our study is the lack of in vivo data, which will be addressed in future. Future studies will also focus on more in-depth mechanisms of the relation between zinc and the regulation of PGC-1α.

Our study shows that infection with MAC significantly induces mitochondrial ROS production, which perpetuates mitochondrial damage, compromising macrophage function. In accordance with this, MAC-infected cells exhibited decreased MMP and physical disruption and loss of integrity of the inner mitochondrial membrane [35], which is required for ATP production. Upon damage to the IMM, the release of ROS and mtDNA triggers mitochondrial-mediated inflammatory signaling, inducing morphological changes that, if unaddressed, lead to cell death. In this study, we reveal that the deleterious effect of MAC upon macrophages was heightened by dietary-induced and genetically induced Zn deficiency in vitro. Importantly, Zn supplementation with a more bioavailable Zn formulation (ZP) attenuated mitochondrial damage and restored macrophage antibacterial function. In related studies, we observed that ZIP8 loss resulted in a significant increase in oxidative-coupled reactions in macrophages in response to bacterial invasion, resulting in decreased bacterial clearance of *Streptococcus pneumoniae*, indicative of dysregulation of oxidative phosphorylation.

These studies are the first to highlight the vital role of Zn in maintaining mitochondrial health in response to MAC infection in macrophages, demonstrating its beneficial impact on PGC-1α. To determine whether Zn impacts PGC-1α-mediated mitochondrial function, we modified culture conditions to emulate dietary-induced Zn deficiency and genetically induced Zn deficiency (ZIP8 loss), and with both conditions, we also supplemented Zn, then evaluated its impact on mitochondrial function and antibacterial activity. Our results consistently revealed an indispensable role of Zn in macrophage function and their ability to eradicate MAC. As this invasive species continues to evolve new mechanisms to evade host defense, we need to aggressively pursue novel, yet tractable, treatments to succeed in this pathogenic arms race. Host-directed therapies designed to enhance natural immune cell function are widely becoming an integral focus in the future of antibacterial treatments. Based on our findings, screening approaches that identify Zn insufficiency due to dietary factors and genetic defects have the potential to foster tolerable preventive and supplemental treatment strategies that more effectively resolve pulmonary MAC infection.

## 4. Materials and Methods

### 4.1. Reagents and Chemicals

The following primers were acquired from Thermo Fisher (Waltham, MA, USA) and used for PCR: *ppargc1* (Mm01208835_m1, hs01016719), *tfam* (Mm00447485_m1, Hs00273372_s1), *gapdh* (Mm99999915_g1, hs02786624). The following primary and secondary antibodies were used: PGC-1α (Cell Signaling Technology (CST), Danvers, MA, USA, 2178), PGC-1α (Santa Cruz Biotechnology (SCBT), Dallas, TX, USA, sc-518025), TFAM (CST, 8076), mtTFA (SCBT, sc-166965), β-Actin (CST, 3700), β-Tubulin (CST, 2146) anti-rabbit IgG HRP (CST, 7074), anti-mouse IgG HRP (CST, 7076), goat anti-rabbit IgG AF 488 (Invitrogen, Waltham, MA, USA, A11008), and donkey anti-mouse IgG AF 647 (Invitrogen, A31571). MitoTracker Green FM (MTG) (Invitrogen, M7514), Tetramethylrhodamine methyl ester (TMRM) (Invitrogen, T668), MitoSOX Red (Invitrogen, M36007) 4’, 6-diamidino-2-phenylindole, dihydrochloride (DAPI) (CST, 4083), Hoechst 33342 (CST, 4082). Unless otherwise specified, reagents and chemicals were obtained through Sigma Aldrich (St. louis, MO, USA) or Thermo Scientific (Waltham, MA, USA).

### 4.2. Animal Use and Care

Animals were maintained at the University of Nebraska Medical Center (UNMC) Animal Facility under pathogen-free conditions, with food and water provided ad libitum. Research protocols were approved by the UNMC Institutional Animal Care and Use Committee (IACUC), under protocols 22–007-04-FC and 16-127. Animal care and procedures were performed in accordance with NIH and Office of Laboratory Animal Welfare (OLAW) Guidelines.

The wildtype (WT) mice used were 4–6-week-old female C57BL/6J (The Jackson Laboratory, Bar Harbor, ME, USA). *Zip8* knockout mice (*Zip8KO*) were generated as previously described [36]. Conditional-ready *Zip8flox/flox* mice from *Zip8flox-neo*/+ mice (C57BL/6NTac-Slc39a8tm1a(EUCOMM)Wtsi/Cnrm) were obtained from the European Mouse Mutant Archive. Heterozygous *Zip8flox-neo*/+ mice were bred to ROSA26:FLPe knock-in mice with ubiquitous expression of FLP1 recombinase (129S4/SvJaeSor- Gt(ROSA)26Sortm1(FLP1) Dym/J; Jackson Laboratory) to delete the Neo cassette adjacent to the upstream loxP site. The resulting *Zip8flox*/+ were mated to produce *Zip8flox/flox* mice. *ZIP8 flox/flox* mice were crossed to myeloid cell specific LysMcre (The Jackson Laboratory) to generate the conditional *Zip8KO* [13,36].

### 4.3. Cell Culture

Human monocyte cell lines U-937 (CRL-1593.2 ATCC, Manassas, VA, USA) and THP-1 (TIB-202, ATCC) were grown in RPMI medium containing 10% fetal bovine serum (FBS) and 1% penicillin–streptomycin [46] and cultured in T75 flasks, with medium changes every other day. Upon reaching confluence, cells were transferred to 15 mL conical tubes and centrifuged at 1300× *g* for 5 min. The medium was aspirated and the cell pellet resuspended in 1 mL of culture medium and filtered through a 70 µm nylon strainer. Cells were counted using a Cell Countess II (Invitrogen) and seeded into plates at the following densities: 6-well 8 × 10^5^, 12-well 4 × 10^5^, 96-well 2.5 × 10^4^. The cells were differentiated into macrophages using PMA [30 ng/mL] for 2 days. Upon differentiation, the PMA-containing medium was aspirated, and the wells were gently washed with warm PBS and incubated with culture medium.

Bone marrow-derived macrophages (BMDMs) were harvested from 4–6-week-old adult C57BL/6J mice (The Jackson Laboratory). Briefly, the mice were anesthetized with isoflurane and sacrificed via cervical dislocation. The hind legs were aseptically dissected, removed, and the femurs were collected in basal RPMI medium and placed on ice. The diaphyses of femurs were cut with scissors, and a 27 G needle and syringe with RPMI were used to flush the bone marrow into a 50 mL conical tube. The bone marrow solution was filtered through a 70 µm nylon filter and centrifuged at 1300× *g* for 6 min at 4 °C. The supernatant was discarded, and ACK lysing buffer (Gibco, Waltham, MA, USA) was added to the cell pellet with gentle pipetting to resuspend. An equal volume of RPMI medium was added, and the cells were recentrifuged. The supernatant was removed, and the cell pellet was resuspended in DMEM culture medium containing mouse M-CSF [50 ng/µL]. The cells were plated in tissue culture dishes (Corning, Corning, NY, USA) and placed in an incubator to allow adherence. The culture medium was changed every other day for 5–7 days until the cells became adherent and confluent. Upon reaching confluence, the medium was aspirated, and the dishes were gently washed with PBS and incubated for 20 min with 0.25% Trypsin. Gentle scraping was used to detach the cells, which were added to a 15 mL conical tube with an equal volume of cell culture medium. The cells were centrifuged, the supernatant was removed, and the pellet suspended in 1 mL of culture medium. The cells were then counted and added to cell culture plates at the following densities: 6-well 1.2 × 10^6^, 12-well 6 × 10^5^, and 96-well 4.5 × 10^4^.

### 4.4. Bacterial Culture

*Mycobacterium avium complex* (MAC) (MAC 101 70089, ATCC) was cultured in liquid 7H9 medium containing 10% OADC (oleic acid, albumin, dextrose, and catalase) supplement and placed in an orbital incubator at 300 RPM at 35 °C for ~48 h until reaching an OD_600_ of ~0.7. Bacterial suspensions were collected and centrifuged at 13,000 RPM for 12 min. The supernatant was discarded, and the pellet was washed in PBS and recentrifuged. The bacterial pellets were then resuspended in PBS, aliquoted into individual tubes at a concentration of 2 × 10^6^ cells/µL, and stored at −80 °C until use [15].

### 4.5. Zinc-Deficient Medium and Zinc Treatment

The Zn-deficient medium was produced through chelation of FBS. Briefly, 500 mL of heat-inactivated FBS was added to a sterile 1 L Erlenmeyer flask containing 100 g of Chelex-100 (BIO-RAD, Hercules, CA, USA), and the solution was mixed overnight at 4 °C. The solution was then filtered, and the Zn-free serum aliquoted into 50 mL conical tubes and stored at −80 °C. The Zn-free serum was used in place of normal FBS in cell culture medium. To induce zinc-deficient conditions, cells were cultured with Zn-free medium for 48 h prior to infection or analysis. Zn treatment was carried out using ZinPRO^®^ (ZP) supplement. ZP was diluted in sterile ddH_2_O and used at a final concentration of 50 µM for 48 h prior to infection or analysis.

### 4.6. Bacterial Infection

Bacteria were cultured and stored as previously noted. Prior to infection, the cell culture medium was replaced with medium containing no antibiotics. MAC cell pellets were thawed and added to the culture medium or directly to wells at an MOI of 1 for either 4 or 24 h [15].

### 4.7. Total RNA Extraction

Cells in 12-well plates (3513, Corning (Corning, NY, USA)) were cultured, treated, and infected, as previously described in Section 4. Total RNA was extracted using the RNeasy Mini Kit (74106, Qiagen, Germantown, MD, USA) according to manufacturer’s instructions. Briefly, cell culture medium was aspirated, wells washed with PBS, and cells lysed and collected through gentle scraping. Lysates were added to RNeasy Mini Spin Columns and briefly centrifuged, with the flow-through discarded. After successive steps of washing and centrifugation with RW1 and RPE buffers, RNA was eluted with nuclease-free water, and the concentration was determined using a NanoDrop One C (Thermo Fisher Scientific, Madison, WI, USA).

### 4.8. RT-PCR

cDNA was synthesized from isolated total RNA using the SuperScript IV First-Strand Synthesis System (18091050, Invitrogen) according to the manufacturer’s instructions. Briefly, kit components and 1 μg of total RNA were added to an 8-tube strip, and reverse transcriptase was performed using a T-100 Thermal Cycler (BIO-RAD).

PCR analysis was performed on a QuantStudio 3 Real-Time PCR system (Thermo Fisher). cDNA was mixed with TaqMan Fast Advanced Master Mix (4444557, Applied Biosystems, San Francisco, CA, USA) and primers in a MicroAmp Optical 96-well Reaction Plate (4306737, Applied Biosystems) and sealed with MicroAmp Optical Adhesive Film (4311971, Applied Biosystems). The quantification of mRNA was determined using the ∆∆CT method.

### 4.9. SDS-PAGE Western BLOT

Cells in 6-well plates (3516, Corning) were cultured, treated, and infected as previously described in Section 4. Cell culture plates were placed on ice, the medium was aspirated, and the wells washed with ice-cold PBS. Cell Lysis Buffer (9803, Cell Signaling Technology (CST)) containing Halt Protease and Phosphatase Inhibitor Cocktail (78440, Thermo Scientific) was added to wells and allowed to gently rock on ice for 15 min. Cell lysates were collected with gentle scraping, transferred to microcentrifuge tubes, centrifuged at 12,000 RPM for 1 min at 4 °C, and briefly sonicated. The lysates were then centrifuged at 12,000 RPM for 12 min at 4 °C, and the supernatant was collected. Protein concentration was determined through comparison with BSA standards using a Pierce Dilution-Free Rapid Gold BCA Protein Assay Kit (A55860, Thermo Scientific) with absorbance read on a BioTek3 Plate Reader (BioTek, Bozeman, MT, USA).

The cell lysates were combined with Laemmli 4X buffer (S3401, Sigma Aldrich) and β-Mercaptoethanol and denatured at 75 °C for 10 min. The reduced lysates were loaded into a 4–15% Mini-PROTEAN TGX SDS PAGE Gel (4561084, BIO-RAD) at a concentration of 30 µg of protein per lane with Precision Plus Protein Kaleidoscope Protein Standards (1610375, BIO-RAD). The gels were resolved in Tris/Glycine/SDS buffer (1610772, BIO-RAD) and run at 125 V for 45 min on a PowerPac HC Power Supply (BIO-RAD). The gels were transferred to PVDF membranes in Tris/Glycine buffer (1610771, BIO-RAD) at 10 V overnight at 4 °C. Transfer efficiency was determined using Ponceau S Stain (P3504, Sigma Aldrich).

Blots were blocked, and primary and secondary antibodies diluted in EveryBlot Blocking Buffer (12010020, BIO-RAD), with extensive washing after each step. The blots were incubated with primary antibodies overnight at 4 °C with gentle rocking. The blots were incubated for 1 h at RT with HRP-conjugated secondary antibody. Protein expression was visualized through the addition of SuperSignal West Femto Maximum Sensitivity Substrate (34094, Thermo Scientific) and imaged on a ChemiDoc MP Imaging System (12003154, BIO-RAD). The blots were stripped using Restore PLUS Western Blot Stripping Buffer (46430, Thermo Scientific) and reprobed with primary and secondary antibodies. Densiometric analysis of bands was performed with ImageJ version 1.51.

### 4.10. Immunocytochemistry

Cells were cultured in 96-well plates (Grenier Bio-One, Monroe, NC, USA), treated and infected as previously detailed. Medium was removed from 96-well plates, and wells were washed with PBS three times for 5 min each. Cells were fixed with PBS containing 4% paraformaldehyde for 15 min, then washed with PBS three times for 5 min each. Cells were then permeabilized with PBS containing 0.3% Triton X-100 for 15 min and washed with PBS three times for 5 min each. PBST containing 5% donkey serum (Gibco) was used for blocking and dilution of antibodies. Cells were blocked for 1 h at room temperature, and solution removed and replaced with buffers containing primary antibodies overnight at 4 °C. Wells were washed with PBS three times for 5 min each, and blocking buffer containing fluorescent secondary antibodies was added to wells for 1 h at room temperature. After removing the solution and washing, cells were counterstained with DAPI [1 µg/mL] for 3 min, then washed again with PBS three times for 5 min each. Cells were then imaged on a BZ-X800 confocal microscope (Keyence, Itasca, IL, USA).

### 4.11. Mitochondrial Staining

Cells in 96-well plates were cultured, treated, and infected, as previously described. Live cell staining was performed using MitoTracker Green (MTG) (Invitrogen) and Tetramethylrhodamine methyl ester perchlorate (TMRM) (Life Technologies, Frederick, MD, USA).

Both fluorescent probes were added to HBSS at a 200 nM concentration for mitochondrial staining for 45 min at 37 °C. The solution was removed, and the wells were gently washed three times with warm HBSS. Cells were counterstained with Hoechst (Cell Signaling Technology) at a concentration of 1 µg/mL in HBSS for 5 min, gently washed, and cell culture medium without phenol red was added to wells. Cells were then imaged on a BZ-X800 confocal microscope (Keyence, Osaka, Japan). Relative fluorescence intensity was determined by averaging fluorescence to the number of DAPI-positive cells.

### 4.12. Bacterial Killing

Quantification of macrophage bactericidal activity was determined through colony counting on agar plates. BMDMs were cultured in 24-well plates and treated as previously described, and cells were infected with MAC at an MOI of 1 for 4 h. The medium was aspirated, the wells were gently washed twice with HBSS, and 100 µL of PBS containing Triton X-100 (1%) and SDS (0.1%) was added to wells. Cell lysates were collected with gentle scraping and serially diluted in PBS to concentrations of 10^−4^, 10^−5^, and 10^−7^. An amount of 50 µL of diluted lysate solution was added to 7H10 agar plates and spread across the plate using an L-shaped cell spreader. Plates were placed in an incubator at 37 °C and 5% CO_2_ atmosphere for 5–7 days, and bacterial colonies were counted and converted to CFU/mL(log) [15].

### 4.13. Phagocytic Uptake

Phagocytic uptake by macrophages was performed using the Vybrant Phagocytosis Assay (Thermo Fisher) according to the manufacturer’s instructions. BMDMs were seeded in 96-well plates and cultured as previously described. The culture medium was aspirated, the wells were washed with PBS, and 200 µL culture medium was added to the wells. The wells were gently scraped and collected for centrifugation (1200× *g* 6 min). Cell counts were determined as previously described, and DMEM was added to each tube to a final concentration of 10^6^ cells/mL. One vial of fluorescent particles and HBSS concentrate was thawed, and 0.5 mL HBSS was added to the particle vial and briefly sonicated to disperse the particles. The suspension was added to a centrifuge tube containing 4.5 mL sterile ddH_2_O, and the mixture was homogenized. Cells were added to a 96-well plate at a density of 1 × 10^5^ cells per well and allowed to adhere for one hour. An amount of 100 µL of the fluorescent particle solution was added to each well and incubated for 2 h. The medium was removed from the wells, and 100 µL of the trypan blue solution was added to quench extracellular fluorescence and allowed to incubate for one minute. The trypan solution was aspirated, and fluorescence (480 nm excitation/520 nm emission) was measured on a microplate reader.

### 4.14. Statistical Analysis

All experiments were performed in triplicate in at least 3 independent experiments, and the data are presented as the mean ± standard error. Data were analyzed for statistical significance using Student’s *t*-test or ANOVA with Excel (Microsoft, Redmond, WA, USA) or PRISM 10.4 (GraphPad, San Diego, CA, USA).

## Data Availability

All relevant data are within the paper or available upon request.
